# A re-analysis of the relationship between “parasite stress” and authoritarianism

**DOI:** 10.3389/fpsyg.2014.00638

**Published:** 2014-06-23

**Authors:** Thomas V. Pollet

**Affiliations:** Department of Social and Organizational Psychology, VU University AmsterdamNetherlands

**Keywords:** parasite stress, pathogen stress, cross-cultural research, political attitudes, authoritarianism, evolutionary psychology

This paper is a commentary on Murray et al. (2013) where, alongside other findings, data from two cross-cultural samples (nations and cultures), supporting a positive relationship between pathogen prevalence and authoritarianism are presented. This commentary is not a theoretical critique (see the comments on Fincher and Thornhill, 2012, for example, or an alternative: Hackman and Hruschka, 2013). Nor is it a methodological critique of work on pathogen stress (e.g., Currie and Mace, 2012; Pollet, 2013; Pollet et al., in press). The goal of this brief commentary is to re-analyze the data with an alternative technique to examine whether the original conclusions are upheld, specifically the claim that indices of pathogen prevalence are positively related to authoritarianism. To this end, I use a relatively novel technique from machine learning: “Conditional inference trees.”

Conditional inference trees are part of “machine learning,” a set of algorithms commonly used for data mining (Hastie et al., 2009 for introduction to field). Researchers may be familiar with machine learning algorithms such as “Classification And Regression Trees” (CART; Breiman et al., 1984) and “CHi-squared Automatic Interaction Detection” (CHAID; Kass, 1980; Biggs et al., 1991). These were originally developed to automatically, and “optimally,” detect interactions and non-linear patterns in data (reviews in Haughton and Oulabi, 1997; Hastie et al., 2009). Next to other limitations, it is known that these “older” algorithms tend to overfit (Hastie et al., 2009). To this end, researchers have recently developed conditional inference trees, which successfully overcome the limitations of these earlier algorithms (Hothorn et al., 2006, 2010; Strobl et al., 2009; Molnar, 2013). Note that these conditional inference trees can be further nested into “Random Forests” (Breiman, 2001; Liaw and Wiener, 2002; Hothorn et al., 2010). Here I focus on, and limit myself to the “ctree” algorithm. As described by Hothorn et al. (2010:8): *“Roughly, the algorithm works as follows: (1) Test the global null hypothesis of independence between any of the input variables and the response (which may be multivariate as well). Stop if this hypothesis cannot be rejected. Otherwise select the input variable with strongest association to the response. This association is measured by a p-value corresponding to a test for the partial null hypothesis of a single input variable and the response. (2) Implement a binary split in the selected input variable. (3) Recursively repeate steps (1) and (2).”* This algorithm does not overfit and no “pruning” is required (Hothorn et al., 2010). Moreover, it demonstrates good properties for regression and classification modeling. Statistical inference is based on permutation testing, which is non-parametric and relatively free of assumptions (Hothorn et al., 2010; Molnar, 2013). A *p*-value can be obtained by comparing the observed data, to the (fully) permuted (randomized) data. The “ctree” algorithm has been extensively tested. It seems particularly useful where researchers are faced with a relatively large number of predictors, which could interact or have non-linear effects, and a relatively small sample, as is the case here. For a full description of the algorithm I refer to the papers cited above.

I apply the “ctree” algorithm from “party” to the datasets provided by Murray et al. (2013). I expanded the first dataset containing countries to incorporate their geographical regions (United Nations, 2013) and their economic development classification (United Nations Development Programme, 2012). Note that this dataset also contained the Human Development index (HDI), which was included in the dataset, but does not seem to be part of the original analyses. The only key change made to second dataset, based on the Standard Cross-Cultural Sample (Murdock and White, 1969), is reversing the sign of “authoritarianism.composite,” which seemed to not have been reverse-coded. I used *all* possible predictors for the algorithm (*n* = 14 and *n* = 25 respectively) and the same outcome variables as Murray et al. (2013). Analyses were run in R version 3.0.1 (R Development Core Team, 2008) in duplicate with different starting seeds. A significance level of 0.05 (corrected for multiple testing) was used. The data used and script are in Supplementary Material.

For the first sample (Figure 1A), the only variable selected was the HDI (*p* = 0.001). The algorithm partitions the data nearly equally, with those countries scoring low on the HDI having high authoritarianism. The pathogen stress variable was not selected by the algorithm as a significant predictor. For the second sample (Figure 1B), the algorithm selected Cashdan and Steele's (2013) pathogen prevalence variable (*p* = 0.002). It implemented a split whereby a cluster (*n* = 26) of countries with low levels of pathogen prevalence also have low authoritarianism. The majority of these cultures (*n* = 15) were North American.

**Figure 1:**
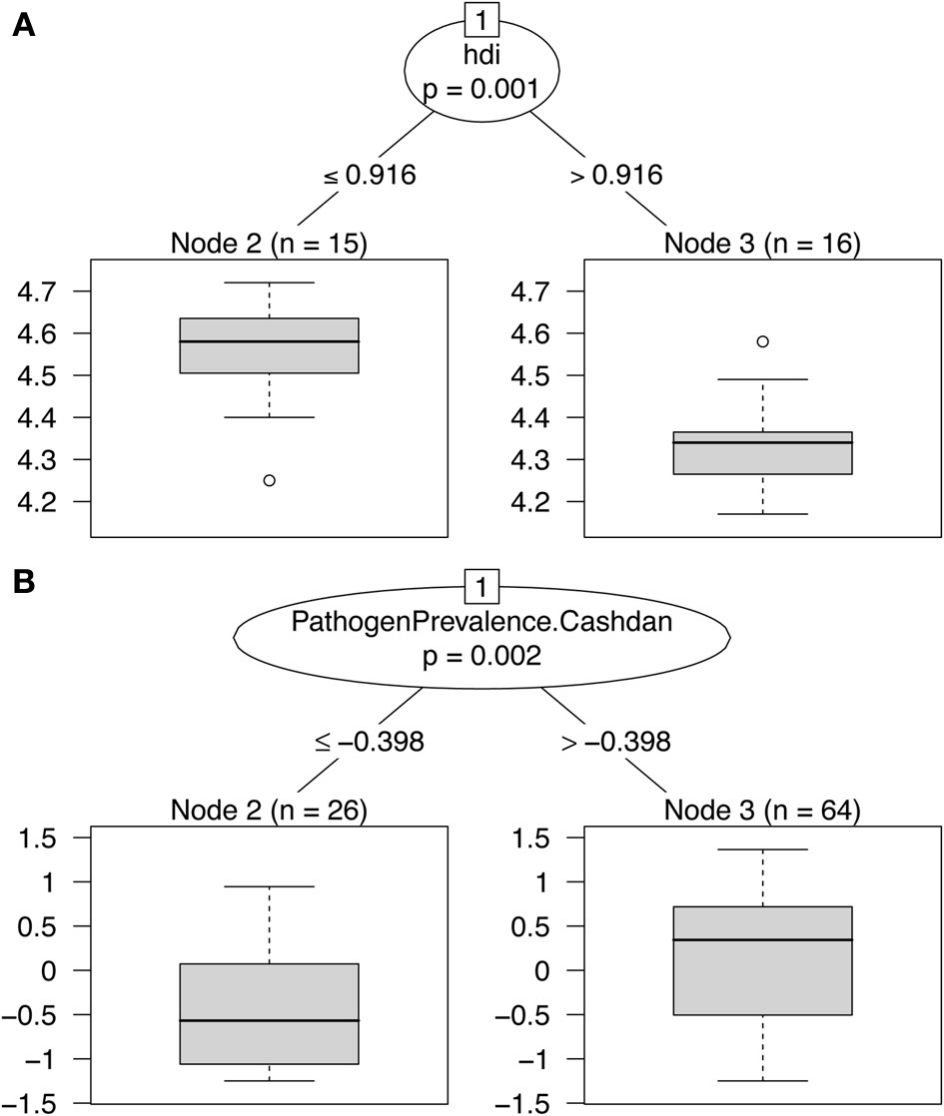
**Results of two conditional inference tree models for Murray et al. (2013)'s data on authoritarianism**. A higher score indicates more authoritarianism. (**A**= Sample 1: 31 Countries, **B**= Sample 2: 90 cultures from the Standard Cross-Cultural Sample).

The current analyses thus provide mixed support for a positive association between pathogen prevalence and authoritarianism. For the first sample, the conclusion is fundamentally different than that of Murray et al. (2013). Only the Human Development Index is found to be a significant predictor of authoritarianism. For the second sample, as in Murray et al. (2013) historical pathogen prevalence seems to be a key predictor. However, it seems that the pattern is non-linear. Rather than an overall linear positive effect of pathogen stress on authoritarianism, as argued by Murray et al. (2013), it appears that there is a cluster of cultures with very low pathogen stress and very low authoritarianism, consisting mainly of North American cultures. This cluster could account for the findings from their correlational and regression analyses.

This alternative approach via conditional inference trees therefore questions the original findings. This alternative technique has some benefits [e.g., good performance with small sample size and multiple (correlated) predictors], but does not overcome some of the limitations inherent to cross-cultural data (e.g., measurement equivalency). Further research examining the effect of pathogen prevalence on politics remains necessary, both at an individual as well as at a cultural level, preferably using advanced statistical techniques. For now, it seems that the purported effects of pathogen stress on authoritarianism at the level of a nation or culture may well hinge upon the analytical strategy, such as correlations and/or ordinary least squares regressions, employed by researchers.

## Conflict of interest statement

The author declares that the research was conducted in the absence of any commercial or financial relationships that could be construed as a potential conflict of interest.
